# Heparin and SARS-CoV-2: Multiple Pathophysiological Links

**DOI:** 10.3390/v13122486

**Published:** 2021-12-11

**Authors:** Pierpaolo Di Micco, Egidio Imbalzano, Vincenzo Russo, Emilio Attena, Vincenzo Mandaliti, Luana Orlando, Maurizio Lombardi, Gianluca Di Micco, Giuseppe Camporese, Saverio Annunziata, Gaetano Piccinocchi, Walter Pacelli, Michele Del Guercio

**Affiliations:** 1 Department of Medicine, Buon Consiglio Fatebenefratelli Hospital of Naples, 80122 Naples, Italy; 2 Dipartimento Di ClinicaMedica E Farmacologia, University of Messina, 98100 Messina, Italy; egidio.imbalzano@unime.it (E.I.); luana_orlando@libero.it (L.O.); 3 Department of Translational Medical Sciences, University of Campania “Luigi Vanvitelli”—Monaldi Hospital, Piazzale Ettore Ruggeri, 80131 Naples, Italy; v.p.russo@libero.it; 4 Division of Cardiology, San Giuliano Hospital, 80014 Giugliano in Campania, Italy; emilio.attena@virgilio.it; 5 Comegen Oupatients Clinic, 80124 Naples, Italy; v.mandaliti@tim.it (V.M.); s.annunziata@libero.it (S.A.); gaetano.piccinocchi@gmail.com (G.P.); 6 Cardiocenter Outpatients Clinic, 80121 Naples, Italy; lombardi.mau@tiscali.it (M.L.); pacelli.walter@gmail.com (W.P.); 7 Division of Cardiology, Ospedale Buon Consiglio, Fatebenefratelli, 80122 Naples, Italy; dimiccomed@gmail.com; 8 Unit of Angiology, Department of Cardiac, Thoracic and Vascular Sciences, Padua University, 35100 Padua, Italy; giuseppe.camporese@aopd.veneto.it; 9 Angiology Outpatients Clinic, 80122 Naples, Italy; micheledelguercio@virgilio.it

**Keywords:** COVID-19, heparins, low molecular weight heparin, fondaparinux, bleedings, venous thromboembolism

## Abstract

Low molecular weight heparin, enoxaparin, has been one of most used drugs to fight the SARS-CoV-2 pandemic. Pharmacological properties of heparin recognize its specific ability, as with other oligosaccharides and glycosaminoglycan, to bind several types of viruses during their pass through the extracellular matrix of the respiratory tract, as well as its anticoagulant activity to prevent venous thromboembolism. Antithrombotic actions of enoxaparin have been testified both for inpatients with COVID-19 in regular ward and for inpatients in Intensive Care Units (ICUs). Prophylactic doses seem to be able to prevent venous thromboembolism (VTE) in inpatients in the regular ward, while intermediate or therapeutic doses have been frequently adopted for inpatients with COVID-19 in ICU. On the other hand, although we reported several useful actions of heparin for inpatients with COVID-19, an increased rate of bleeding has been recorded, and it may be related to several conditions such as underlying diseases with increased risks of bleeding, increased doses or prolonged administration of heparin, personal trend to bleed, and so on.

## 1. Background 

Heparin and heparan sulphate are complex, linear, acidic polysaccharides belonging to the glycosaminoglycan (GAG) family; their structure makes continuous alternating glucosamine and uronic acid units; for these reasons, they are considered polyanions [1]. The continued sequence of uronic acids and amino sugars has ester sulphates and that the amino groups were (partly) acetylated [1]. Its structure is like heparin sulphate [2]. Physiologically, heparin and heparan sulphate are the main constituents of the extracellular matrix, and heparin is exactly released in granules of mast cells [1,2,3]. The anti-protease mechanism’s function [4] gives evidence of its importance, helping to regulate several tissues’ function during normal state and during disease states. Heparin has an anti-protease action that can reduce the activity of numerous proteases of the clotting system; its anticoagulant power, in fact, is well known and testified in several reports in vitro and in vivo [5]. 

The anticoagulant activity of heparin is due to its property to bind an anti-protease of the serpin family named antithrombin III (ATIII) [4]; this binding greatly accelerates the ability of ATIII to inactivate several clotting proteases, such as thrombin (factor IIa) and prothrombinases (factor Xa) [6,7].

From a pharmacological point of view, we distinguish two different types of heparins: unfractionated heparins (UFH) and low molecular weight heparins (LMWH) [8]. UFH is a heterogenous preparation of anionic, sulphated glycosaminoglycan polymers with high weights, ranging from 3 kDa to 30 kDa, while LMWH have a reduced weight that ranges from 4.5 kDa to 10 kDa [8]. A different weight is derived from different enzymatic depolymerization of the whole heparin and a different weight also has a different pharmacokinetic effect, because it may induce a more selective inhibition of factor Xa for LMWH, versus a more selective action to inhibit factor IIa by UFH [9]. Yet, the anti-inflammatory effect of LMWH is also different because flow weight fragments are less able to bind proteases and cells in the extracellular matrix [10]. 

**Figure 1:**
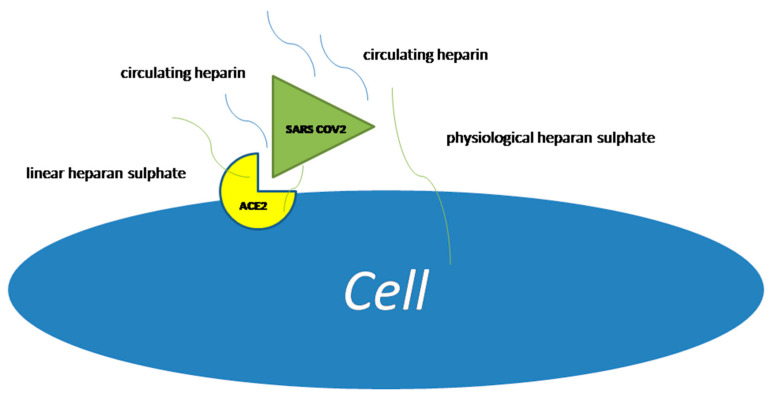
Heparan sulphate binding with SARS-CoV-2 and angiotensin converting. Enzyme receptor type 2 (ACE2).

On the other hand, heparan sulphate, the main component at cell surfaces and in the extracellular matrix of most animal tissues, can interact with a large variety of protein and cells, so takes a relevant role in disease processes. Most functions of heparan sulphate depend on ionic interactions, with a variety of proteins, including growth factors, cytokines, and their receptors [10]. Furthermore, heparan sulphate is also able to interact with SARS-CoV-2 before its binding to angiotensin converting enzyme receptor type 2 (ACE-2) (Figure 1). Several studies have confirmed that these mechanisms are effective for SARS-CoV-2, and that SARS-CoV-2 spike protein interacts with both cellular heparan sulphate and angiotensin-converting enzyme 2 (ACE2) through its receptor [11].

## 2. Heparin and SARS CoV2

Coronaviruses (CoVs) are single-stranded RNA viruses which originate from a well-known family of viruses named Coronaviridae [12]. Usually, Coronavirus affects animals such as cows, pigs and birds. When the virus infects humans, respiratory infections are possible, and they vary from less severe respiratory or gastrointestinal disease to severe acute respiratory syndrome (SARS) [13,14]. One of the known epidemics of SARS, due to a Coronavirus, and named SARS-CoVs, has been certified in 2003, and another epidemic hotbed due to a Coronavirus was identified in 2012 in the Middle East; for this reason, the induced respiratory infection was named Middle East Respiratory Syndrome (MERS) [14]. In the last 2 years, a pandemic due to another Coronavirus, i.e., SARS-CoV-2, began in China and then diffused all around the world. The virus as known is able to induce a multifocal pneumonia with possible lung failure [15]. The disease was named COVID-19 by World Health Organization (WHO).

Since the first epidemiological report from different cohorts of patients affected by COVID-19 in China, this infection has been associated to hypercoagulable state and thrombotic complications [16,17]. For this reason, all scientific societies suggested pharmacological thromboprophylaxis with low molecular weight heparin for all inpatients with COVID-19. 

Yet, before exerting its specific actions, SARS-CoV-2 binds several types of glycosaminoglycans and oligosaccharides, such as fucosylated acid, sialic acid and heparan sulphate [18].

Heparan sulphate is abundant in the respiratory tract, and it plays a role as binding factor for coronaviruses with tropism to bronchitis and other respiratory infections [19]. For this reason, these actions may explain in part the extended tropism of this virus for cells of respiratory tract, and the similarity between heparan sulphate and heparin gives further protective actions to heparin itself.

## 3. Prophylactic Doses of Low Molecular Weight Heparin in Inpatients with COVID-19

As previously reported, COVID-19 predisposes patients to venous thromboembolism (VTE), due to excessive inflammation, platelet activation, and endothelial dysfunction. The association between COVID-19 and VTE is well known since first reports from China, and this association took clinical relevance, because the association between these two conditions was associated to a worse prognosis in Chinese population [20].

Yet, thrombotic risk seems to be different in patients with different intensity of care [21]: patients admitted to the ICU, in fact, show an increased trend to develop VTE compared to those admitted in regular ward [22,23]. Additional risk factors for death or death for associated VTEin several cohorts are diabetes or other cardiovascular risk factors [24,25].

For this reason, pharmacological prophylaxis to prevent VTE has been suggested since the beginning of pandemic, to escape thrombotic complications for inpatients [26].

Prophylactic doses of low molecular weight heparin have been reported as safe regarding the prevention of VTE in inpatients in regular ward [27]. Furthermore, the rate of bleeding in inpatients treated with prophylactic doses of LMWH was also low, as already reported [27,28].

Yet, the duration of thromboprophylaxis with standard doses of low molecular weight heparin after discharge is still matter of discussion; in particular, the administration of LMWH is considered to be safe, since the first signs and symptoms of the disease and during the hospitalization, while the long term prophylaxis also after hospital discharge did not report univocal consensus for the relevant outcomes (i.e., rate of VTE, rate of bleeding, overall death), as far as for the duration of treatment. 

## 4. Intermediate and Therapeutic Doses of Low Molecular Weight Heparin in Inpatients with COVID-19

The high risk of developing VTE in patients with SARS-CoV-2 has raised hypotheses about the use of intermediate and therapeutic doses of LMWH, which have not yet developed events and therefore in primary prevention.

As shown in literature by clinical trials as ATTACC [29], REMAP-CAP [30], enoxaparin at therapeutic doses as an initial strategy would reduce thromboembolic events in patients with a not advanced state of disease [31], despite no significant major bleeding recorded. 

In the HEP-COVID trial [32], in patients with not critic disease, D-dimer levels greater than 4 times the normal or a sepsis-induced coagulopathy (SIC) score ≥4, LMWH has significantly reduced the risk of the primary endpoint, the composite of three events: VTE, arterial thrombosis events (ATE) and all-cause mortality (ACM), with a negligible hemorrhagic risk. Therefore, the clinical conditions of patients in a mild or initial state of disease are straightforward, to allow the use of the therapeutic dose of enoxaparin, reducing the composite of death and VTE, compared to the standard thromboprophylaxis doses. 

The case for ICU patients is different, in which the treatment with therapeutic doses with LMWH appeared not superior to the usual-care doses, for primary prevention of the onset of VTE [33]. In addition, at the base of the not optimal effectiveness of low molecular weight heparin in this type of patients, there would be a reduced subcutaneous absorption due to increased vascular permeability, the resulting edema and increased vascular distribution with reduced oncotic pressure [34].

To date, no doubts should be raised about the use of anticoagulant therapy in patients with COVID-19, having guaranteed a good safety profile in prophylactic and therapeutic doses.

According to the literature [35], reduced efficacy of heparin in therapeutic doses in the advanced state of illness, induces to evaluate the component time as the best element in the therapeutic strategy, starting treatment as soon as possible, exposing the patient to the numerous pleiotropic effects of the molecule, in an early stage of the disease.

## 5. Fondaparinux and COVID-19

The depolymerization of heparin results not only in the development of LMWHs but also in the synthesis of fondaparinux, that is composed only of the pentasaccharidic sequence of heparin that is able to bind antithrombin III with a specific propensity to selective inhibition of Xa more than IIa [36]; from a pharmacodynamics point of view, it does not interact with plasma proteins other than antithrombin, leading to a predictable pharmacokinetics [37].

The clinical utility of fondaparinux in VTE preventions has been reported in several clinical contexts as major orthopaedic surgery and acute medical illness [38,39].

So, as COVID-19 is an acute infection that required hospitalization in (majority) most of the cases of prolonged hospitalization, an adequate thromboprophylaxis with fondaparinux has been suggested and performed in several studies [40,41]. A reduced rate of VTE events was found in the Italian cohort in which Fondaparinux 2.5 mg daily was compared to Enoxaparin 4000 I.U. or 6000 I.U. daily [42,43], while an increased anti-inflammatory action of enoxaparin was found in another study from the same group [44]. In another study, by another Italian group, a small increase in bleedings was recorded in inpatients with prophylactic doses of fondaparinux [41].

Because these cohorts of patients were no larger, the benefit of the clinical utility of Fondaparinux 2.5 mg as thromboprophylaxis for inpatients with COVID-19 was postponed in all reported studies to other scientific research on a large population. 

## 6. Bleedings in Inpatients with COVID-19 Treated with Low Molecular Weight Heparin

The risk of bleeding should always be considered when an antithrombotic drug is administered to a patient, both if prophylactic or therapeutic doses are considered [45]. Several bleeding risk scores are suggested in the literature to quick estimate this risk [46]. Yet, during COVID-19 pandemic, alerts given on the great prothrombotic effect of infection per se and on the relevant risk to develop VTE for inpatients in ICU or in regular ward induced to consider intermediate or therapeutic doses of LMWH to prevent VTE, although randomized trials were not available during the first waves of the pandemic.

Although the risk of VTE has been confirmed also by several scientific societies, the use of therapeutic doses to prevent VTE has been frequently discouraged by experts [47].

In a multicenter study from the USA, in fact, for the first time, it has been reported that the rate of bleeding for inpatients with COVID-19 was really similar to the rate of VTE, so therapeutic doses were not suggested by Authors and a clinical input to a right surveillance of bleedings was suggested in their conclusion [48]. Regarding the evaluation of bleeding risk factors in this study, the use of LMWH and increased levels of d-dimer at admission in hospital were found as atypical but predictive risk factors for bleeding. 

In another large series from the RIETE registry, the incidence of bleeding increased nearly 5.0%, and a great part of patients received thromboprophylaxis with intermediate or therapeutic doses of LMWH [49].

These data testify that clinically, the risk of bleeding should be always considered, although prothrombotic diseases such as COVID-19 show an increased risk of fatal pulmonary embolism.

## 7. Inside Anti-Inflammatory and Antithrombotic Actions of Heparins

Since WHO suggested the use of heparins during the pandemic of SARS-CoV-2, a large series of studies regarding protective action of low molecular weight heparin in inpatients has been reported. Standard doses, intermediated doses and therapeutic doses of low molecular weight heparin have been tested in several cohorts around the world, with positive outcomes regarding VTE.

Yet, heparins may also exert their pharmacological activity in other ways. Besides its action toward serinproteases of clotting system as factor IIa and/or Xa by its pentasaccharide motif, heparin may exert its anti-inflammatory action toward other proteases nexin I, as far as human neutrophil elastases and others by its arginine residues, and mainly OSO3- groups [50]. For these reasons, heparin showed its therapeutic effect in acute or subacute inflammatory diseases as pancreatitis, as respiratory inflammations (e.g., COVID-19 and lung emphysema), as far as inflammatory bowel disease [51,52,53,54]. 

## 8. Low Molecular Weight Heparin in Patients with COVID-19 Treated at Home

In about 15% of COVID-19 patients, the clinical course of the disease may be complicated by the onset of severe interstitial pneumonia, with possible lung failure that may require hospitalization; however, many infected patients remain asymptomatic or paucisymptomatic, and are managed in outpatient settings [55,56].

Following the clinical indication of WHO [57], Italian Minister of Health by the Italian Society of General Medicine confirmed the suggestion to the use of prophylactic doses of LMWH, because of the possible hypomobility, also for not-hospitalized patients because of quarantine and because of treatment of hypoxia at home. Of course, an evaluation of bleeding risk should be performed in any case, being a prophylactic treatment suggested until the nasopharyngeal swab was negative and/or until the hypomobility was absent (e.g., 15 or 30 days).

Furthermore, a following study on patients treated at home for COVID-19 revealed that LMWH was preferred by several experts for patients with associated cardiovascular comorbidities, with low rates of pulmonary complications, or need of hospitalization for lung failure [58].

Moreover, a further study from another Italian group, that treated patients with COVID-19 at home with high flow nasal cannulae, revealed that thromboprophylaxis with standard doses of LMWH are suggested, in any case, to escape vascular complication as pulmonary embolism at home. 

## 9. Conclusions

The two-way link between the inflammation and clotting system is well established and based on several mechanisms, as reduction of the activity of natural anticoagulants as far as inhibition of fibrinolysis, and the pro-inflammatory and pro-coagulant action of cytokines by increasing expression of tissue factor on monocytes and endothelium is established, thus giving a hyperactivation of the clotting cascade [59,60]. The role of heparin to improve the outcome of patients with COVID-19 has been underlined since the first wave of the outbreak, and reinforced in these months, because the associated reduced mortality independently from the modification of inflammatory biomarkers [61]. Improvements reported with the use of heparins have been also underlined in Guidelines of several scientific societies [62]; for this reason, other anticoagulants do not appear as suggested anticoagulants to prevent VTE in inpatients with COVID-19, although some cohorts reported good results [63,64].

Together to its well know anti-thrombotic role, that is needed to reduce the rate of thrombotic complications, heparin showed further useful actions in vitro and in vivo.

Anti-inflammatory effects have been reported in several series and in experimental model with the reduction of cytokines [65], and a specific anti-viral action in extracellular matrix of tissues has also been described.

So, the use of heparin is suggested, since the first clinical signs of confirmed infection by SARS-CoV-2, although the majority of positive effects have been demonstrated for inpatients vs. patients treated at home; furthermore, heparin use is suggested also after discharge from hospital as for other acute medical illnesses. Yet, overdose of heparin with intermediated or therapeutic doses in patients with COVID-19 are also associated to similar rates of thrombotic and hemorrhagic complications that may be associated with worse outcomes.

## Data Availability

Not applicable.

## References

[1] Shriver Z., Capila I., Venkataraman G., Sasisekharan R. (2012). Heparin and heparansulfate: Analyzing structure and microheteroge-neity. Heparin-A Century Prog..

[2] Li J.P., Kusche-Gullberg M. (2016). HeparanSulfate: Biosynthesis, Structure, and Function. Int. Rev. Cell Mol. Biol..

[3] Mulloy B., Lever R., Page C.P. (2016). Mast cell glycosaminoglycans. Glycoconj. J..

[4] Mulloy B., Hogwood J., Gray E., Lever R., Page C.P. (2015). Pharmacology of Heparin and Related Drugs. Pharmacol. Rev..

[5] Wardrop D., Keeling D. (2008). The story of the discovery of heparin and warfarin. Br. J. Haematol..

[6] Rezaie A.R., Giri H. (2020). Anticoagulant and signaling functions of antithrombin. J. Thromb. Haemost..

[7] Schoen P., Lindhout T., Willems G., Hemker H.C. (1989). Antithrombin III-dependent anti-prothrombinase activity of heparin and heparin fragments. J. Biol. Chem..

[8] Hirsh J. (1992). Overview of low molecular weight heparins and heparinoids: Basic and clinical aspects. Aust. N. Z. J. Med..

[9] Boneu B., Dol F., Caranobe C., Sie P., Houin G. (1989). Pharmacokinetics of heparin and related polysaccharides. Ann. N. Y. Acad. Sci..

[10] Xu D., Esko J.D. (2014). Demystifying heparansulfate-protein interactions. Annu. Rev. Biochem..

[11] Clausen T.M., Sandoval D.R., Spliid C.B., Pihl J., Perrett H.R., Painter C.D., Narayanan A., Majowicz S.A., Kwong E.M., McVicar R.N. (2020). SARS-CoV-2 Infection Depends on Cellular Heparan Sulfate and ACE. Cell.

[12] Siddell S.G., Anderson R., Cavanagh D., Fujiwara K., Klenk H.D., Macnaughton M.R., Pensaert M., Stohlman S.A., Sturman L., van der Zeijst B.A. (1983). Coronaviridae. Intervirology.

[13] Jung K., Saif L.J., Wang Q. (2020). Porcine epidemic diarrhea virus (PEDV): An update on etiology, transmission, pathogenesis, and prevention and control. Virus Res..

[14] de Wit E., van Doremalen N., Falzarano D., Munster V.J. (2016). SARS and MERS: Recent insights into emerging coronaviruses. Nat. Rev. Microbiol..

[15] Zhou F., Yu T., Du R., Fan G., Liu Y., Liu Z., Xiang J., Wang Y., Song B., Gu X. (2020). Clinical course and risk factors for mortality of adult inpatients with COVID-19 in Wuhan, China: A retrospective cohort study. Lancet.

[16] Tang N., Li D., Wang X., Sun Z. (2020). Abnormal coagulation parameters are associated with poor prognosis in patients with novel coronavirus pneumonia. J. Thromb. Haemost..

[17] Klok F.A., Kruip M.J.H.A., van der Meer N.J.M., Arbous M.S., Gommers D.A.M.P.J., Kant K.M., Kaptein F.H.J., van Paassen J., Stals M.A.M., Huisman M.V. (2020). Incidence of thrombotic complications in critically ill ICU patients with COVID-19. Thromb. Res..

[18] Di Micco P., Di Micco G., Russo V., Poggiano M.R., Salzano C., Bosevski M., Imparato M., Fontanella L., Fontanella A. (2020). Blood Targets of Adjuvant Drugs Against COVID 19. J. Blood Med..

[19] Madu I.G., Chu V.C., Lee H., Regan A.D., Bauman B.E., Whittaker G.R. (2007). Heparansulfate is a selective attachment factor for the avian coronavirus infectious bronchitis virus Beaudette. Avian Dis..

[20] Wang D., Hu B., Hu C., Zhu F., Liu X., Zhang J., Wang B., Xiang H., Cheng Z., Xiong Y. (2020). Clinical Characteristics of 138 Hospitalized Patients With 2019 Novel Coronavirus-Infected Pneumonia in Wuhan, China. JAMA.

[21] Canoğlu K., Şaylan B., Çalışkan T. (2021). COVID-19 and thrombosis: Prophylaxis and management. Tuberk. Ve Toraks.

[22] Mattioli M., Benfaremo D., Mancini M., Mucci L., Mainquà P., Polenta A., Baldini P.M., Fulgenzi F., Dennetta D., Bedetta S. (2020). Safety of intermediate dose of low molecular weight heparin in COVID-19 patients. J. Thromb. Thrombolysis.

[23] Di Micco P., Tufano A., Cardillo G., Imbalzano E., Amitrano M., Lodigiani C., Bellizzi A., Camporese G., Cavalli A., De Stefano C. (2021). The Impact of Risk-Adjusted Heparin Regimens on the Outcome of Patients with COVID-19 Infection. A Prospective Cohort Study. Viruses.

[24] Zhu N., Zhang D., Wang W., Li X., Yang B., Song J., Zhao X., Huang B., Shi W., Lu R. (2020). A Novel Coronavirus from Patients with Pneumonia in China. N. Engl. J. Med..

[25] Klok F.A., Kruip M.J.H.A., van der Meer N.J.M., Arbous M.S., Gommers D., Kant K.M., Kaptein F.H.J., van Paassen J., Stals M.A.M., Huisman M.V. (2020). Confirmation of the high cumulative incidence of thrombotic complications in critically ill ICU patients with COVID-19: An updated analysis. Thromb. Res..

[26] Cuker A., Tseng E.K., Nieuwlaat R., Angchaisuksiri P., Blair C., Dane K., Davila J., DeSancho M.T., Diuguid D., Griffin D.O. (2021). American Society of Hematology 2021 guidelines on the use of anticoagulation for thrombo-prophylaxis in patients with COVID-19. Blood Adv..

[27] Thoreau B., Galland J., Delrue M., Neuwirth M., Stepanian A., Chauvin A., Dellal A., Nallet O., Roriz M., Devaux M. (2021). D-Dimer Level and Neutrophils Count as Predictive and Prognostic Factors of Pulmonary Embolism in Severe Non-ICU COVID-19 Patients. Viruses.

[28] Musoke N., Lo K.B., Albano J., Peterson E., Bhargav R., Gul F., DeJoy R Salacup G., Pelayo J., Tipparaju P., Azmaiparashvili Z. (2020). Anticoagulation and bleeding risk in patients with COVID-19. Thromb Res..

[29] Houston B.L., Lawler P.R., Goligher E.C., E Farkouh M., Bradbury C., Carrier M., Dzavik V., A Fergusson D., A Fowler R., Galanaud J.-P. (2020). Anti-Thrombotic Therapy to Ameliorate Complications of COVID-19 (ATTACC): Study design and methodology for an international, adaptive Bayesian randomized controlled trial. Clin. Trials.

[30] Angus D.C., Berry S., Lewis R.J., Al-Beidh F., Arabi Y., Van Bentum-Puijk W., Bhimani Z., Bonten M., Broglio K., Brunkhorst F. (2020). The REMAP-CAP (Randomized Embedded Multifactorial Adaptive Platform for Community-acquired Pneumonia) Study. Rationale and Design. Ann. Am. Thorac. Soc..

[31] Lawler P.R., Goligher E.C., Berger J.S., Neal M.D., McVerry B.J., Nicolau J.C., Gong M.N., Carrier M., Rosenson R.S., Reynolds H.R. (2021). Therapeutic Anticoagulation with Heparin in Noncritically Ill Patients with Covid-19. N. Engl. J. Med..

[32] Spyropoulos A.C., Goldin M., Giannis D., Diab W., Wang J., Khanijo S., Mignatti A., Gianos E., Cohen M., Sharifova G. (2021). Efficacy and Safety of Therapeutic-Dose Heparin vs. Standard Prophylactic or Intermediate-Dose Heparins for Thromboprophylaxis in High-risk Hospitalized Patients With COVID-19. JAMA Intern. Med..

[33] REMAP-CAP, ACTIV-4a and ATTACC Investigators (2021). Therapeutic Anticoagulation with Heparin in Critically Ill Patients with Covid-19. N. Engl. J. Med..

[34] Zufferey P.J., Dupont A., Lanoiselée J., Bauters A., Poissy J., Goutay J., Jean L., Caplan M., Levy L., Susen S. (2021). Pharmacokinetics of enoxaparin in COVID-19 critically ill patients. Thromb. Res..

[35] Al-Samkari H., Gupta S., Leaf R.K., Wang W., Rosovsky R.P., Brenner S.K., Hayek S.S., Berlin B.H., Kapoor R., Shaefi S. (2021). Thrombosis, Bleeding, and the Observational Effect of Early Therapeutic Anticoagulation on Survival in Critically Ill Patients With COVID-19. Ann. Intern. Med..

[36] Gerotziafas G.T., Petropoulou A.D., Verdy E., Samama M.M., Elalamy I. (2007). Effect of the anti-factor Xa and anti-factor IIa activities of low-molecular-weight heparins upon the phases of thrombin generation. J. Thromb. Haemost..

[37] Bauer K.A. (2001). Fondaparinux sodium: A selective inhibitor of factor Xa. Am. J. Heal. Pharm..

[38] Falck-Ytter Y., Francis C.W., Johanson N.A., Curley C., Dahl O.E., Schulman S., Ortel T.L., Pauker S.G., Colwell C.W. (2012). Prevention of VTE in Orthopedic Surgery Patients: Antithrombotic Therapy and Prevention of Thrombosis, 9th ed: American College of Chest Physicians Evidence-Based Clinical Practice Guidelines. Chest.

[39] Kahn S.R., Lim W., Dunn A.S., Cushman M., Dentali F., Akl E.A., Cook D.J., Balekian A.A., Klein R.C., Le H. (2012). Prevention of VTE in nonsurgical patients: Antithrombotic Therapy and Prevention of Thrombosis, 9th ed: American College of Chest Physicians Evidence-Based Clinical Practice Guidelines. Chest.

[40] Russo V., Proietti R., Lodigiani C., Di Micco P. (2021). Fondaparinux and bleeding risk in COVID-19: Unsolved question. Thromb. Res..

[41] Prandoni P., Cattelan A.M., Carrozzi L., Leone L., Filippi L., De Gaudenzi E., Villalta S., Pesavento R. (2020). The hazard of fondaparinux in non-critically ill patients with COVID-19: Retrospective controlled study versus enoxaparin. Thromb. Res..

[42] Russo V., Cardillo G., Viggiano G.V., Mangiacapra S., Cavalli A., Fontanella A., Agrusta F., Bellizzi A., Amitrano M., Iannuzzo M. (2020). Thromboprofilaxys with Fondaparinux vs. Enoxaparin in Hospitalized COVID-19 Patients: A Multicenter Italian Observational Study. Front. Med. (Lausanne).

[43] Russo V., Cardillo G., Viggiano G.V., Mangiacapra S., Cavalli A., Fontanella A., Agrusta F., Bellizzi A., Amitrano M., Iannuzzo M. (2020). Fondaparinux Use in Patients With COVID-19: A Preliminary Multicenter Real-World Experience. J. Cardiovasc. Pharmacol..

[44] Cardillo G., Viggiano G.V., Russo V., Mangiacapra S., Cavalli A., Castaldo G., Agrusta F., Snr A.B., Amitrano M., Iannuzzo M. (2021). Antithrombotic and Anti-Inflammatory Effects of Fondaparinux and Enoxaparin in Hospitalized COVID-19 Patients: The FONDENOXAVID Study. J. Blood Med..

[45] Depietri L., Marietta M., Scarlini S., Marcacci M., Corradini E., Pietrangelo A., Ventura P. (2018). Clinical impact of application of risk assessment models (Padua Prediction Score and Improve Bleeding Score) on venous thromboembolism, major hemorrhage and health expenditure associated with pharmacologic VTE prophylaxis: A “real life” prospective and retrospective observa-tional study on patients hospitalized in a Single Internal Medicine Unit (the STIME study). Intern. Emerg. Med..

[46] Piovella C., Dalla Valle F., Trujillo-Santos J., Pesavento R., López L., Font L., Valle R., Nauffal D., Monreal M., Prandoni P. (2014). Comparison of four scores to predict major bleeding in patients receiving anticoagulation for venous throm-boembolism: Findings from the RIETE registry. Intern. Emerg. Med..

[47] Bikdeli B., Madhavan M.V., Jimenez D., Chuich T., Dreyfus I., Driggin E., Nigoghossian C., Ageno W., Madjid M., Guo Y. (2020). COVID-19 and Thrombotic or Thromboembolic Disease: Implications for Prevention, Antithrombotic Therapy, and Follow-Up: JACC State-of-the-Art Review. J. Am. Coll. Cardiol..

[48] Al-Samkari H., Karp Leaf R.S., Dzik W.H., Carlson J.C.T., Fogerty A.E., Waheed A., Goodarzi K., Bendapudi P.K., Bornikova L., Gupta S. (2020). COVID-19 and coagulation: Bleeding and thrombotic manifestations of SARS-CoV-2 infection. Blood.

[49] Fernández-Capitán C., Barba R., Díaz-Pedroche M.D.C., Sigüenza P., Demelo-Rodriguez P., Siniscalchi C., Pedrajas J.M., Farfán-Sedano A.I., Olivera P.E., Gómez-Cuervo C. (2020). Presenting Characteristics, Treatment Patterns, and Outcomes among Patients with Venous Thromboembolism during Hospitalization for COVID-19. Semin. Thromb. Hemost..

[50] Hornebeck W., Lafuma C., Robert L., Móczár M., Móczár E. (1994). Heparin and its Derivatives Modulate Serine Proteinases (SERPS) Serine Proteinase Inhibitors (SERPINS) Balance: Physiopathological Relevance. Pathol.-Res. Pract..

[51] Tozlu M., Kayar Y., Ince A.T., Baysal B., Senturk H. (2020). Low molecular weight heparin treatment of acute moderate and severe pancreatitis: A randomized, controlled, open-label study. Turk. J. Gastroenterol..

[52] Redini F., Lafuma C., Hornebeck W., Choay J., Robert L. (1988). Influence of heparin fragments on the biological activities of elastase(s) and α1 proteinase inhibitor. Biochem. Pharmacol..

[53] Hippensteel J.A., LaRiviere W.B., Colbert J.F., Langouët-Astrié C.J., Schmidt E.P. (2020). Heparin as a therapy for COVID-19: Current ev-idence and future possibilities. Am. J Physiol. Lung Cell Mol. Physiol..

[54] Day R., Forbes A. (1999). Heparin, cell adhesion, and pathogenesis of inflammatory bowel disease. Lancet.

[55] Russo V., Piccinocchi G., Mandaliti V., Annunziata S., Cimmino G., Attena E., Moio N., Di Micco P., Severino S., Trotta R. (2020). Cardiovascular Comorbidities and Pharmacological Treatments of COVID-19 Patients Not Requiring Hospitalization. Int. J. Environ. Res. Public Health.

[56] Luks A.M., Swenson E.R. (2020). Pulse Oximetry for Monitoring Patients with COVID-19 at Home. Potential Pitfalls and Practical Guidance. Ann. Am. Thorac. Soc..

[57] Home Care for Patients with COVID-19 Presenting with Mild Symptoms and Management of Their Contacts: Onterim Guidance. https://www.scribd.com/document/452851700/WHO-nCov-IPC-HomeCare-2020-3-eng.

[58] Barco S., Bingisser R., Colucci G., Frenk A., Gerber B., Held U., Mach F., Mazzolai L., Righini M., Rosemann T. (2020). Enoxaparin for primary thromboprophylaxis in ambulatory patients with coronavirus disease-2019 (the OVID study): A structured summary of a study protocol for a randomized controlled trial. Trials.

[59] Maduzia D., Ceranowicz P., Cieszkowski J., Chmura A., Galazka K., Kusnierz-Cabala B., Warzecha Z. (2020). Administration of warfa-rin accelerates the recovery in ischemia/reperfusion-induced acute pancreatitis. J. Physiol. Pharmacol..

[60] Warzecha Z., Sendur P., Ceranowicz P., Dembinski M., Cieszkowski J., Kusnierz-Cabala B., Tomaszewska R., Dembinski A. (2015). Pre-treatment with low doses of acenocoumarol inhibits the development of acute ischemia/reperfusion-induced pancreatitis. J. Physiol. Pharmacol..

[61] Pereyra D., Heber S., Schrottmaier W.C., Santol J., Pirabe A., Schmuckenschlager A., Kammerer K., Ammon D., Sorz T., Fritsch F. (2021). Low molecular weight heparin use in COVID-19 is associated with curtailed viral persistence—A retrospective multicenter observational study. Cardiovasc. Res..

[62] Moores L.K., Tritschler T., Brosnahan S., Carrier M., Collen J.F., Doerschug K., Holley A.B., Jimenez D., Le Gal G., Rali P. (2020). Prevention, diagnosis, and treatment of VTE in patients with coronavirus disease 2019: CHEST guideline and expert panel report. Chest.

[63] Wenzler E., Engineer M.H., Yaqoob M., Benken S.T. (2020). Safety and Efficacy of Apixaban for Therapeutic Anticoagulation in Criti-cally Ill ICU Patients with Severe COVID-19 Respiratory Disease. TH Open.

[64] Russo V., DIMaio M., Attena E., Silverio A., Scudiero F., Celentani D., Lodigiani C., Di Micco P. (2020). Clinical impact of pre-admission antithrombotic therapy in hospitalized patients with COVID-19: A multicenter observational study. Pharmacol. Res..

[65] Litov L., Petkov P., Rangelov M., Ilieva N., Lilkova E., Todorova N., Krachmarova E., Malinova K., Gospodinov A., Hristova R. (2021). Molecular Mechanism of the Anti-Inflammatory Action of Heparin. Int. J. Mol. Sci..

